# Cherubism Unmasked: A Case Report of Clinical and Histopathological Presentation

**DOI:** 10.7759/cureus.56456

**Published:** 2024-03-19

**Authors:** Sakshi Akolkar, Alka Hande, Archana M Sonone, Ankita Chavhan, Husna Tehzeeb

**Affiliations:** 1 Oral Pathology and Microbiology, Sharad Pawar Dental College and Hospital, Datta Meghe Institute of Higher Education & Research, Wardha, IND

**Keywords:** osteoblastic rimming, eosinophilic cuffing, multinucleated gaint cell, fibrous dysplasia, fibro-osseous lesions, cherubism

## Abstract

Cherubism, an uncommon genetic disorder, manifests as painless swelling in both jaws. A 20-year-old male presented with symmetrical swelling in both the mandible and maxilla. The jaws exhibited bilateral expansion, typical of this condition. Dentofacial abnormalities associated with cherubism stem from mutations in the SH3BP2 gene, which plays a crucial role in regulating osteoblasts and osteoclasts. In summary, cherubism is a genetic disorder characterized by non-cancerous jaw bone lesions. Surgical intervention may be necessary for functional or aesthetic concerns.

## Introduction

Fibro-osseous lesions (FOLs) encompass a range of conditions that impact the jaws and bones of the face and skull, showcasing the diversity within this category. These lesions are defined by the replacement of normal osseous tissue of the bone with fibrocellular tissue along the areas of mineralization, which can differ in size and appearance [1]. The FOLs of the maxillofacial bones are benign spindle cell proliferations that contain varied amounts of woven bone. Several distinct entities have been proposed based on histological and radiographical traits [2]. These sets of lesions are recognized to share some common clinical, radiologic, and histological characteristics. Despite having similar characteristics, they represent significant diagnostic and treatment issues for clinicians and pathologists, which leads to a particular treatment approach [3]. However, because there is a significant overlap in histologic features across these lesions, the complicated nomenclature can be reduced by classifying lesions based on their radiographical features [2].

WHO classified FOLs in 2005 as (1) ossifying fibroma (OF); (2) fibrous dysplasia (FD); (3) osseous dysplasia (OD); (4) central giant cell granuloma (CGCG); (5) cherubism; (6) aneurysmal bone cyst; and (7) solitary bone cyst. OF necessitates complete removal from the surrounding bone due to its increased risk of recurrence. Conversely, FD is managed conservatively because it tends to resolve on its own [4].

Cherubism is an uncommon, benign, self-limiting genetic disorder primarily impacting the jaws during childhood and adolescence. It is characterized by fibro-osseous changes. It is evident in the third to fourth decade of life [5]. The first case of cherubism was reported in 1952. The term “cherubism” comes from the word “cherub,” which refers to angels with childish, wide-cheeked faces gazing upward, as if their eyes are looking up to “heaven” [6,7]. It is mostly observed in children ages 0-15. It is an autosomal dominant condition that mostly affects males [8]. This state is marked by a progressive growth of cysts in the mandible and maxilla, accompanied by their spontaneous breakdown and subsequent bone regeneration during puberty [5]. Initially considered a form of craniofacial FD specific to the jaws, it is presently identified as a distinct disorder with its own genetic cause [9]. Clinical findings include familial incidents, both sides of jaw involvement in early childhood, elevated palate, missing teeth, especially second and third molars, abrupt cessation or regression of lesions after attainment of puberty, and one of the characteristic features is the rare involvement of the temporomandibular joint [5].

Also, there is a painless bilateral expansion of the maxillary and mandibular bones, which results in a moon face and a heavenward gaze. The mandible is more commonly affected than the maxilla in cherubism. In some cases, initial presentations may involve lymph node enlargement, which contributes to the characteristic cherubic appearance [9]. As the fibrocellular metaplastic lesion progresses, protruding masses may extend into the floor of the orbits, leading to the characteristic upward tilting of the eyes and revealing the sclera below the iris [10]. Quiescent cherubism lesions are commonly found in older individuals and typically show minimal progression. They may diminish over time, and surgical intervention is recommended primarily for cosmetic purposes or to alleviate discomfort resulting from compression effects [11]. Thus, radiological and histopathological characteristics are the mainstays for the diagnosis of cherubism.

## Case presentation

A 20-year-old male presented to the outpatient department of oral and maxillofacial surgery at our hospital with a chief complaint of swelling over the right and left sides of the face and jaw for one year. The patient had apparently been alright eight years ago, after which he had begun experiencing swelling over the bilateral sides of his face and jaw. The swelling was initially small and had increased to the present size gradually. There were no reported incidents of trauma or the discharge of pus. The patient provided a history of tooth exfoliation in the lower front region of the jaw. Additionally, there was a history of blurred vision from the left eye for two to three years and mild paresthesia over the right side of the nose. There was no significant medical or family history.

During the physical examination, the patient appeared robust, energetic, and mentally attentive. No abnormalities were detected upon clinical assessment of the chest, abdomen, cardiovascular system, or central nervous system. There were no signs of skin pigmentation or other congenital anomalies, and no indications of endocrine disorders were observed.

**Figure 1:**
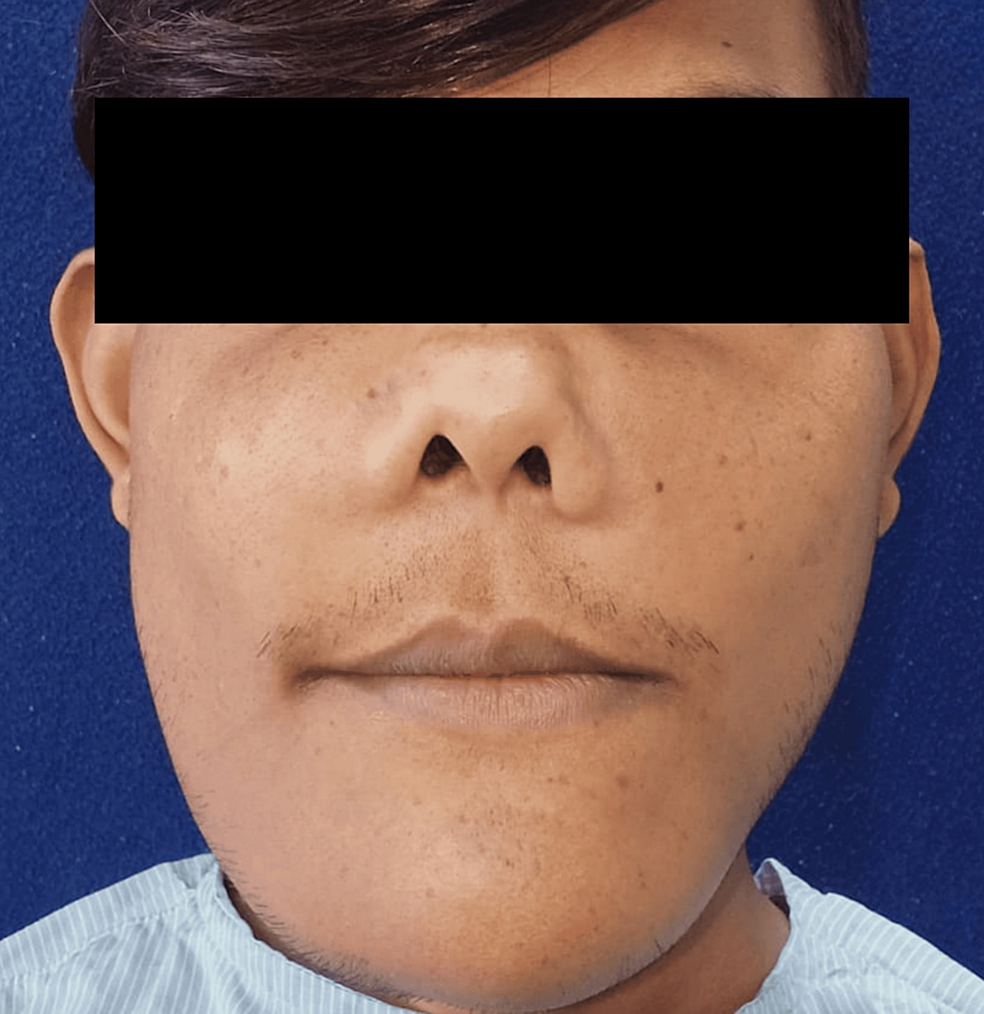
Clinical presentation showing swelling over the angles of the mandible, extending to the malar bones on both sides

**Figure 2:**
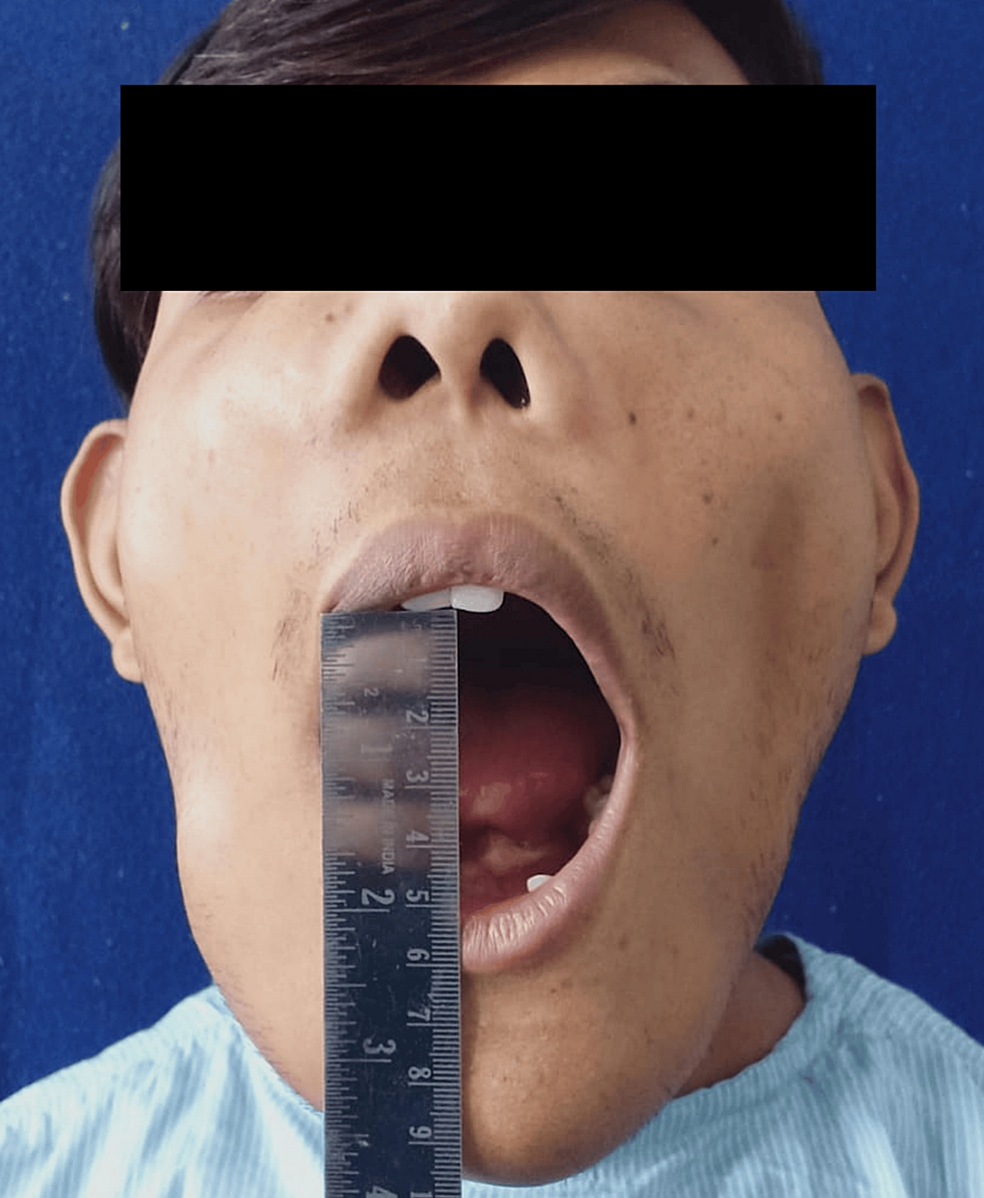
Clinical presentation showing adequate mouth opening

**Figure 3:**
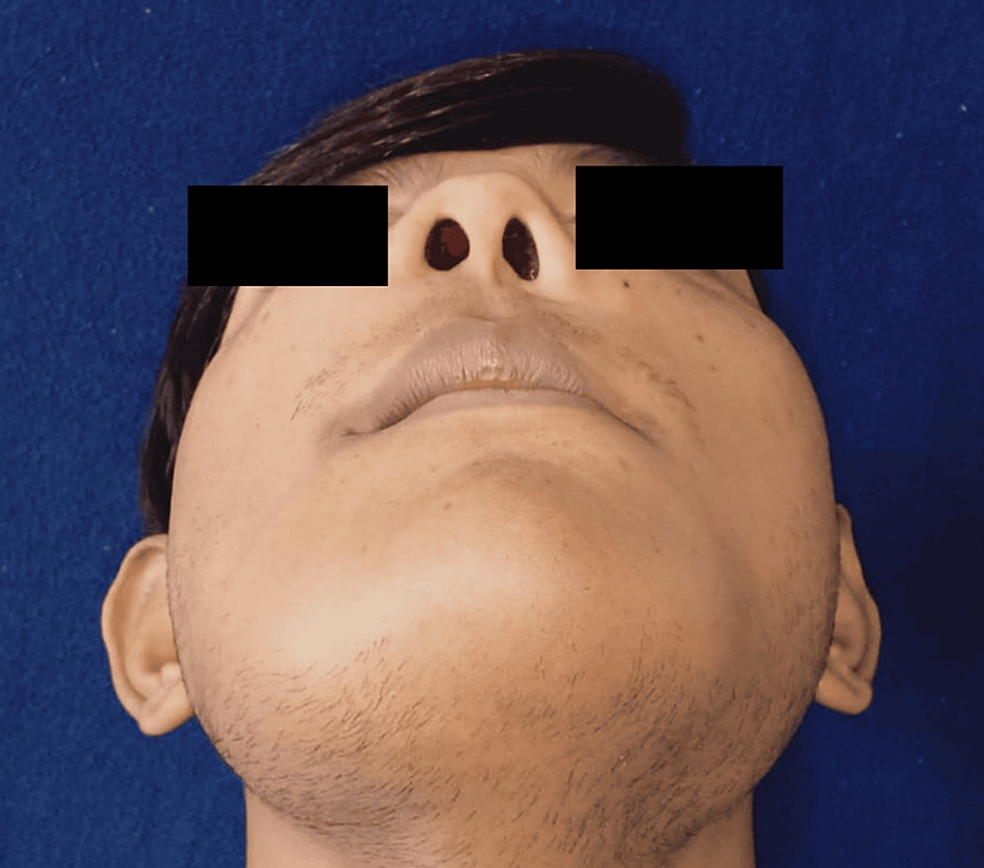
Clinical presentation showing bilateral submandibular swelling

During the extraoral examination, diffuse swelling was observed over the angles of the mandible, extending to the malar bones on both sides (Figure 1). The skin covering the swelling appeared normal. Upon palpation, the swelling was non-painful and exhibited a hard consistency suggestive of bone involvement, with no localized increase in temperature. Bilaterally, palpable submandibular lymph nodes were seen, which were non-painful and movable. Intraoral examination revealed missing teeth 12, 13, 22, 31, 32, 33, 41, and 42 (Figure 2, Figure 3). Mild gingival inflammation was noted.

**Figure 4:**
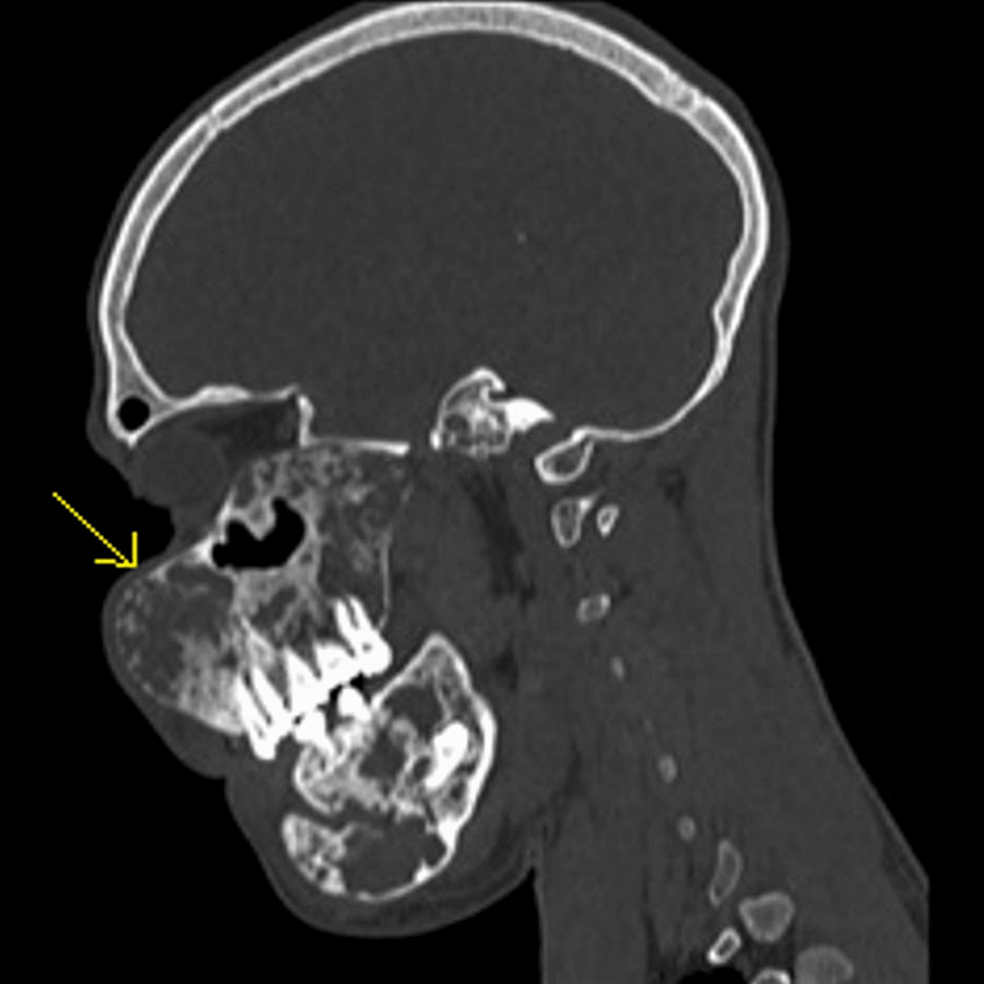
The sagittal section of a reconstructed CT scan of the facial bones reveals an expansile lesion with osteolytic characteristics and a slight ground glass appearance present in both the right upper and lower jaw

**Figure 5:**
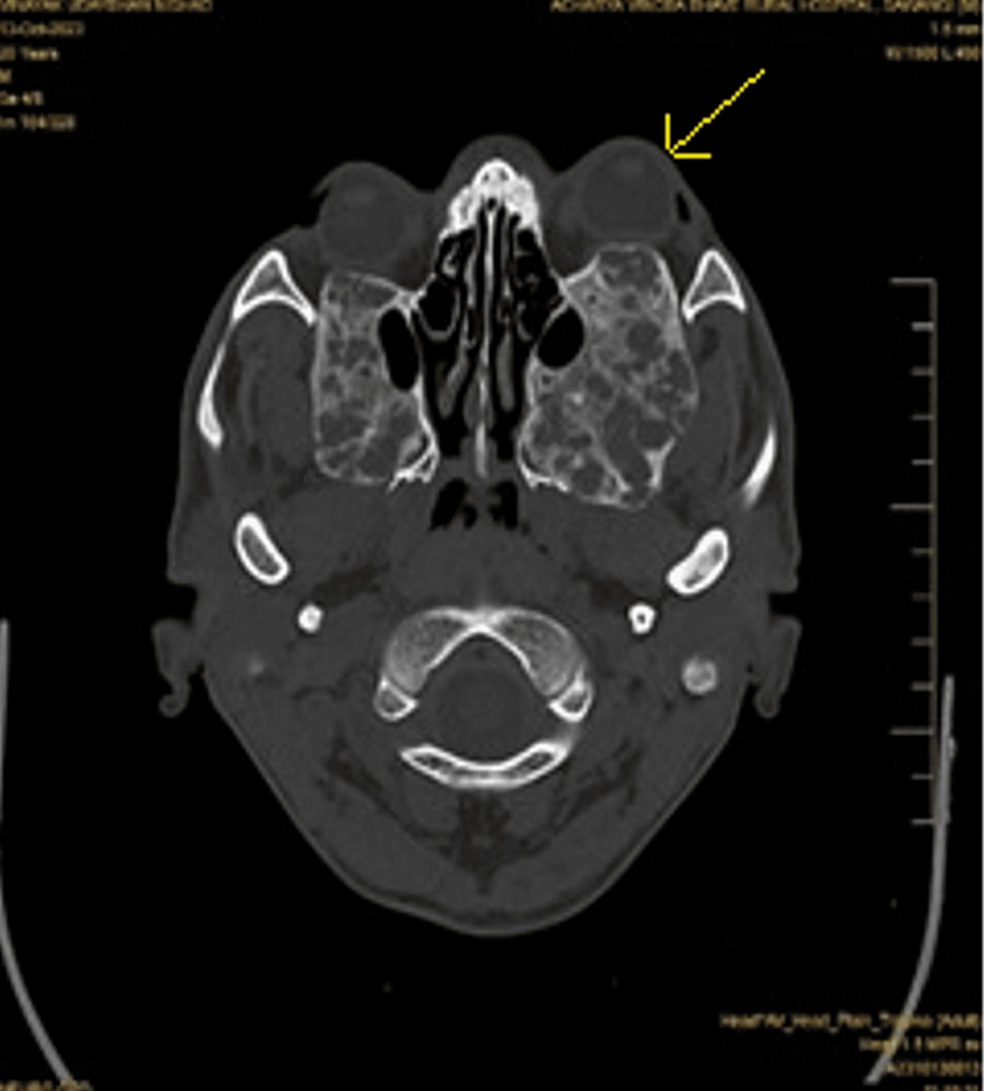
Axial section of CT showing expansion and multilocularity of the lesions

X-rays, CT scans, and MRIs were suggested to confirm the diagnosis, assess the extent of bone involvement, and plan treatment. CT facial bones on radiological evaluation revealed diffuse extraoral expansion of bone calvarium with an intact cortex and loss of trabeculae, giving a homogenous ground glass appearance noted in the mandible and maxilla (Figure 4). There was involvement of the bilateral floor of the orbits, bilateral pterygoid plates, and maxillary sinuses, with partial obscuration of the sinuses. Bilateral pyriform, epiglottis folds, thyroid cartilage, esophagus, and trachea appeared normal (Figure 5). Mucosal thickening was noted in the maxillary sinus.

**Figure 6:**
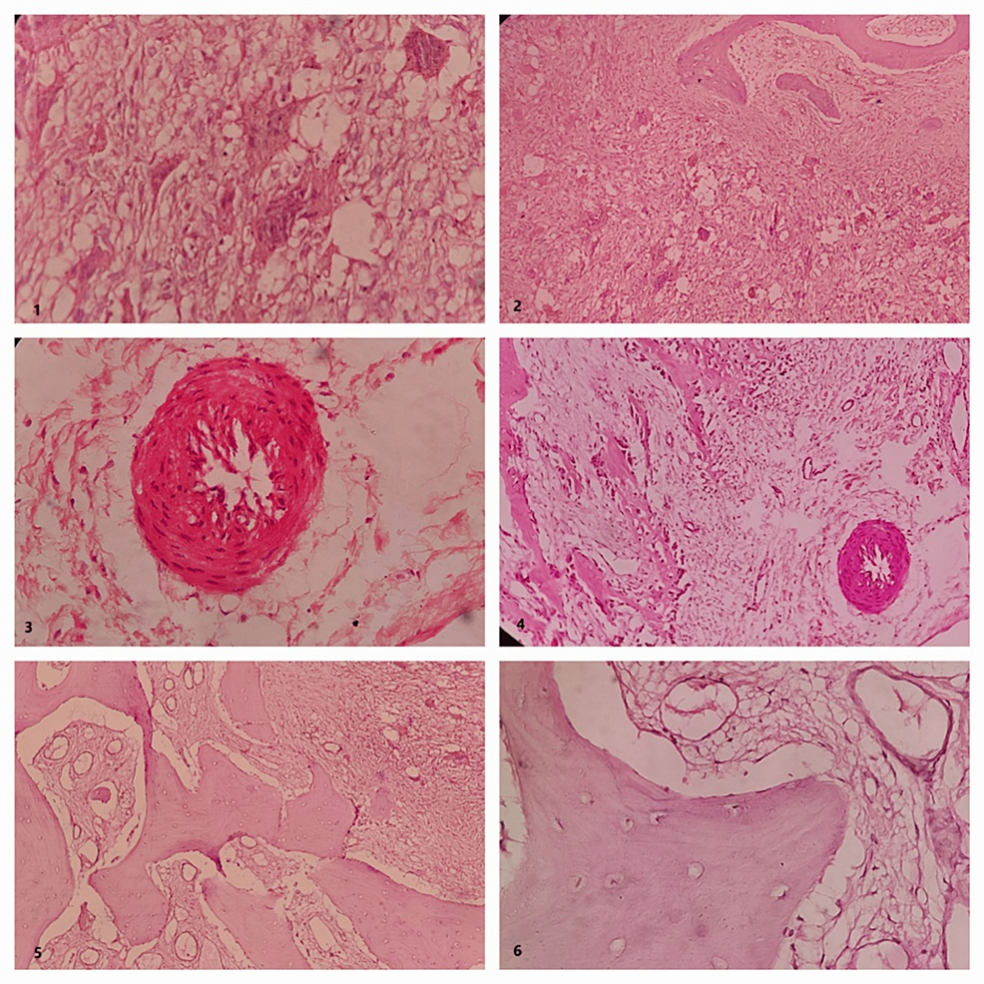
(1) Multinucleated giant cells. (2) Bony trabaculae with giant cells. (3) Perivascular eosinophilic cuffing. (4) Bony trabaculae with perivascular eosinophilic cuffing. (5) Bony trabaculae with osteocytes. (6) Bony trabeculae with osteoblastic rimming

The clinical and radiological presentation led to the provisional diagnosis of cherubism. A bilateral incisional biopsy was carried out, and histopathological examination revealed loosely organized fibrocellular connective tissue accompanied by numerous multinucleated giant cells containing seven to 10 nuclei. The giant cells tended to be small and aggregate focally. Eosinophilic cuffing appeared to be significantly present. Also, H&E-stained sections showed numerous bony trabeculae, along with osteoblastic rimming and the presence of numerous osteocytes entrapped. Cherubism was conclusively diagnosed by considering the clinical, radiographic, and histopathological features (Figure 6).

## Discussion

FOLs encompass various conditions marked by the substitution of typical bone with fibrous tissue along with a newly formed mineralized substance. Common examples of FOLs comprise FD, cemento-OD, OF, and cherubism [8]. The cause and underlying pathophysiology of FD in bones remain unidentified and poorly understood [12]. FD is a progressive, tumor-like condition characterized by the substitution of healthy bone with an abundance of fibrous connective tissue containing irregular trabeculae [8]. There is no preference based on gender, and the femur, tibia, cranial bones, and ribs are the most frequently affected areas [13]. Diagnosing the subtypes of FD requires a thorough evaluation, considering two main factors: (a) the extent of skeletal involvement, distinguishing between monostotic and polyostotic forms, and (b) the presence of any extraskeletal manifestations [14]. The primary anomaly in FD stems from a postnatal mutation occurring in the GNAS1 gene. This mutation prompts the spread and initiation of undifferentiated mesenchymal cells, interrupting bone growth during the woven phase and leading to their transformation into fibro-osseous cystic tissue. Cherubism, on the other hand, represents a genetic variant of FD triggered by mutations in the autosomal dominant SH3BP2 gene. This condition manifests in two distinct forms: a milder, localized monostotic form and a more aggressive polyostotic form [12,15]. Cherubism is an uncommon, non-neoplastic hereditary condition that causes painless bilateral jaw swelling, causing paresthesia [5]. It is an uncommon large-cell disease of the bone that occurs in children. Several family accounts from several countries have identified cherubism as a genetic bone condition [6]. Cherubism is a bone disorder that typically emerges during childhood and is characterized by the development of bilateral and symmetrical proliferative FOLs, primarily affecting the jaw and maxilla. There is a symmetrical expansion. The phenotype of cherubism varies widely, ranging from being clinically asymptomatic to severe mandibular and maxillary overgrowth, which can lead to issues with respiration, vision, speech, and swallowing [5]. The differential diagnosis of cherubism includes FD of the jaws, CGCG, brown tumor, true giant cell tumor, and infantile hyperostosis [6].

Cherubism ranges according to its severity and is classified into the following categories: (1) inactive lesions are typically found in adolescence and are non-progressive; (2) non-aggressive lesions are typically seen at a younger age and grow extremely slowly; and (3) aggressive lesions appear in childhood, expand quickly, and can cause tooth mobility, resorption of roots, and weakening of the cortical bone. A surgical approach may be required to treat the condition [12]. The present case falls under the category of inactive lesions.

Depending on histological, immune, histochemical, and structural characteristics, Chomette and colleagues described cherubism in three stages: the first stage features many multinucleated giant cells. These cells show positive activity for tartrate-resistant acid phosphatase. These tissues are filled with blood vessels. The lesions’ periphery contains a high concentration of fibroblastic cells. Endothelial cells show hemosiderin pigments. The second stage, the reparative stage, is characterized by proliferating spindle cells. The main features observed include prominent fibroblastic nodules surrounding central vessels. The presence of a newly formed bony matrix and osteoid is clearly noticeable. The third stage is the formation of osteoids, which is observed alongside the mineralizing matrix.

They possess greater collagen content but fewer cells. The large, multinucleated cells are recognized as osteoclasts. The elongated fibroblast-like cells are referred to as fibroblasts or myofibroblast cells. Metabolically active juvenile fibroblasts are characterized by ovoid shapes. The bilateral maxilla exhibits a ground-glass appearance [11]. Histopathological examination showed numerous giant cells of different sizes along with varying numbers of cores in the mesenchymal stroma background, a characteristic finding consistent with cherubism [11]. The classification and grading of cherubism by Motamedi (1998) and Raposo-Amaral (2007) are presented in Table 1 [16,17].

**Table 1:** Classification and grading of cherubism by Motamedi and Raposo-Amaral

Grade I: Lesions with the mandible involved, but there are no signs of root resorption	
Subtypes	Description
Subtype 1	Body of the mandible with a single lesion present.
Subtype 2	Body of the mandible with more than one lesions.
Subtype 3	Mandibular ramus with a single lesion present.
Subtype 4	Lesions involving the ramus region of both sides.
Subtype 5	Lesions on the body of the mandible, including the ramus.
Grade II: Involvement of maxillary and mandibular bone with multiple lesions without resorption of the root of teeth	
Subtypes	Description
Subtype 1	A lesion is present posteriorly in the upper and lower jaws.
Subtype 2	Lesions are present anteriorly in the upper and lower jaws.
Subtype 3	Lesions are present in the lower jaw and the entire upper jaw.
Grade III: Involvement of mandibular bone with invasive lesions along with resorption of the root of teeth	
Subtypes	Description
Subtype 1	Body of the mandible with a single lesion present.
Subtype 2	Body of the mandible with more than one lesion.
Subtype 3	Mandibular ramus with a single lesion present.
Subtype 4	Lesions involving the ramus region of both sides.
Subtype 5	Lesions on the body of the mandible and ramus regions of both sides.
Grade IV: Lesions involving the mandible and maxilla and showing signs of resorption of the root of teeth	
Subtypes	Description
Subtype 1	A lesion is present posteriorly in the upper and lower jaws.
Subtype 2	Lesions are present anteriorly in the maxilla and mandible.
Subtype 3	Lesions are present in the mandible and entire maxilla.
Grade V: It is the juvenile kind, which is uncommon, rapidly developing, invasive, and can disfigure. Lesions affecting the maxilla and mandible, including the coronoid and condyles	
Grade VI: It is extremely rare, rapidly growing in size, invasive, and causes deformity	

## Conclusions

While relatively rare, cherubism exerts a significant impact on patients and their families due to the development of facial deformities, functional challenges, and social well-being concerns. The majority of cherubism cases resolve spontaneously without requiring surgical intervention. However, in cases where cherubism rapidly progresses, surgical resection may be necessary. Despite the challenges associated with cherubism, advancements in diagnostic techniques and treatment modalities offer hope for improved outcomes and quality of life for affected individuals. Long-term follow-up is crucial to monitor disease progression, assess treatment efficacy, and manage potential complications effectively.
